# Revealing the structure and distribution changes of *Eucalyptus* lignin during the hydrothermal and alkaline pretreatments

**DOI:** 10.1038/s41598-017-00711-w

**Published:** 2017-04-04

**Authors:** Chenzhou Wang, Hanyin Li, Mingfei Li, Jing Bian, Runcang Sun

**Affiliations:** 1 Beijing Key Laboratory of Lignocellulosic Chemistry, Beijing Forestry University, Beijing, 100083 China; 2 State Key Laboratory of Pulp and Paper Engineering, South China University of Technology, Guangzhou, 510640 China

## Abstract

An integrated pretreatment process based on hydrothermal pretreatment (HTP) followed by alkaline pretreatment has been applied to treat *Eucalyptus*. The chemical composition and structure changes of lignin during the pretreatment were comprehensively characterized. The surface morphology of the cell walls and lignin distribution of the pretreated *Eucalyptus* were detected by scanning electron and confocal Raman microscopies. It was found that the chemical bonds between lignin and hemicelluloses were cleaved during the pretreatment. The results also indicated that the contents of *β*-*O*-4′, *β*-*β*′, and *β*-5′ linkages were decreased with the increase of hydrothermal pretreatment temperature and the cleavage of *β*-*O*-4′ linkages in lignin was accompanied with repolymerization reactions. ^31^P NMR analysis showed that the content of aliphatic OH was reduced as the temperature increased and the total phenolic OH was elevated and then declined with the increase of temperature. Raman spectra analysis revealed that the dissolution rate of lignin in the secondary wall regions was faster than that in cell corner middle lamella regions during the pretreatment. These results will enhance the understanding of the cell wall deconstruction during the pretreatment and the mechanism of the integrated pretreatment process acting on *Eucalyptus*.

## Introduction

With the ever-increasing energy consumption and deterioration of environment around the world, the exploitation of renewable resources for the production of fuels and materials has garnered much attention. Lignocellulosic biomass, such as wood, grass, agricultural and forestry residues, is the most precious renewable resource of the natural world because it can effectively store solar energy^1–3^. Biofuels produced from biomass are feasible to replace petroleum due to their renewability, inexpensiveness, and environmental friendliness^4^. *Eucalyptus* is a promising forestal resource for biomass production due to its rapid growth rate, adaptability, and efficiency in biomass accumulation^5, 6^. However, the natural recalcitrance of the plant cell wall is the critical obstacle of the high efficient conversion of lignocelluloses to biofuels. The natural recalcitrance is a structural and chemical property of biomass for inhibiting deconstruction on its cell walls, which originates not only from the composition of the cell wall polymers (cellulose, hemicelluloses and lignin), but also from fine details of their macromolecular structure and conformation, and on their highly ordered architecture at scales from a few nanometers to several microns^2, 7^. Therefore, an effective and cost-competitive pretreatment technology is required as the first step to reduce the biomass recalcitrance.

To date, a series of pretreatment technologies including physical, chemical, physicochemical and biological approaches have been proposed to overcome the biomass recalcitrance^4, 8^. Among these pretreatments, hydrothermal pretreatment (HTP) is an economical and environmentally friendly pretreatment technology for lignocelluloses, since it uses only feedstock and water as the medium, avoiding corrosion problems and the formation of neutralization sludges^9^. HTP can release hemicelluloses and less lignin from materials, together with some chemicals, such as furfural, formic, acetic, and levulinic acids^10^. Moreover, physical disruption of the lignocellulose structure induced by HTP results in decreased crystallinity and DP of cellulose, which can enhance the availability of cell wall polysaccharides to enzymatic access^11, 12^. However, some limitations restrict the application of HTP, for instance, only partial removal of hemicelluloses and incomplete disruption of lignin–hemicelluloses matrix. Therefore, the combination of a further pretreatment with HTP is required to achieve a better performance for biomass pretreatment. It has been reported that alkaline pretreatment is a promising technology for the disruption of cell wall by solubilizing hemicelluloses and lignin effectively^13^. During the alkaline pretreatment, the alkali-labile linkages in hemicelluloses and lignin are easily broken down, resulting in the exposure of cellulose to enzymes.

Lignin is regarded as one of the main factors that affect the production of biofuels by impeding the enzymatic hydrolysis. In general, lignin is a complex three-dimensional phenyl-propanoid polyphenol inside the cell wall and the main functions of lignin are to provide rigidity and physical strength to the plants, to keep water and nutrients in the fibers, and to protect plants from biological attack^14^. Lignin is usually biosynthesized by three aromatic alcohol precursors (monolignols): coniferyl, sinapyl, and *p*-coumaryl alcohols, which give raise to guaiacyl (G), syringyl (S), and *p*-hydroxyphenyl (H) units respectively in the lignin structure^15^. The monolignols are linked together via radical coupling reactions to form a complex three-dimensional molecular architecture that contains a great variety of bonds, such as ether (*β*-*O*-4′, *α*-*O*-4′, and 4-*O*-5′) and C–C (*β*-*β*′, *β*-5′, and *β*-1′) interunit linkages^15, 16^. Besides the linkages within lignin itself, lignin is known to bind physically/chemically to cellulose and hemicelluloses by covalent bonds. Hence, it is necessary to investigate the structural changes of lignin during the pretreatment to advance the production of both biofuels and bio-materials. Moreover, the information of the spatial distribution of lignin in plant cell walls is crucial to further understand the mechanism of the pretreatment. Due to the development of laser and detector technologies, confocal Raman microscopy (CRM) has been widely applied to give insights into both the chemical and structural information of lignocellulosic materials *in situ* on the subcellular level^17, 18^.

The aim of the present study was to investigate the detailed information on the structure and spatial distribution changes of lignin during the two-step pretreatment consisting of hydrothermal and alkaline pretreatments. The chemical analyses combined with microscopic imaging techniques were used to monitor visually the course of the delignification during the pretreatment. The structural and physicochemical features of the lignin fractions were investigated by high performance anion exchange chromatography (HPAEC), gel permeation chromatography (GPC), semi-quantitative two-dimensional heteronuclear essential single quantum coherence (2D-HSQC), and ^31^P NMR spectroscopy. The surface morphology of the plant cell walls and the spatial distribution of lignin were investigated by scanning electron microscopy (SEM) and CRM. The dynamic information acquired by CRM would greatly enhance the understanding of the cell wall deconstruction during the pretreatment.

## Results and Discussion

### Yields and Sugar Analysis of the Lignin Fractions

Table 1 shows the HTP residue yields of hydrothermal residues, the yields of lignin fractions (based on the initial weight of the raw material) and the contents of its associated carbohydrates. As shown, the HTP residue yields were affected by the pretreatment severity and decreased from 73.2 to 61.2%, which was mainly due to the degradation of hemicelluloses and the solubilization of amorphous cellulose during hydrothermal pretreatment. The hydrothermal pretreatment of the *Eucalyptus* samples at 170, 180, 190, 200, and 210 °C for 0.5 h combined with the alkaline pretreatment with 2% NaOH at 80 °C for 2 h resulted in a dissolution of 3.3, 5.1, 5.8, 7.5, and 2.7% of the lignin fractions, respectively. It was worth noting that the yield of the lignin fraction L_170_ (3.3%) was lower than that of L_0_ (4.5%), which might be due to the mild destruction of the fiber matrix resulting from the degraded lignin under low temperature during the hydrothermal pretreatment^19^. The yields of the lignin fractions increased from 5.1% to 7.5% as the temperature increased from 180 to 200 °C, indicating that the chemical bonds between lignin and hemicelluloses were cleaved to some extent. Additionally, it seemed that the lignin was migrated and relocated to a more localized and concentrated distribution within the cell wall during the hydrothermal pretreatment, thus leading to the easier isolation of lignin by the following alkaline extraction^20, 21^. However, the yield of the lignin fraction decreased to 2.7% when the temperature was further raised to 210 °C. The reduced yield was probably due to the formation of pseudo-lignin by the combination of carbohydrates and lignin degradation products under the harsh HTP conditions^22^.

**Table 1:** HTP residue yields and contents of associated carbohydrates of the lignin fractions obtained from *Eucalyptus*.

Lignin fraction^a^	Yield^b^	Associated sugar						HTP residue yield^e^ (%)
		Ara^c^	Gal^c^	Glc^c^	Xyl^c^	Man^c^	Total sugars^d^	
L_0_	4.5	0.04	0.03	0.04	0.03	0.01	0.15	100.0
L_170_	3.3	0.02	0.01	0.05	0.23	ND^f^	0.32	73.2
L_180_	5.1	ND	ND	0.03	0.13	ND	0.16	67.1
L_190_	5.8	ND	ND	0.03	0.11	ND	0.14	66.8
L_200_	7.5	ND	ND	0.06	0.04	ND	0.10	64.9
L_210_	2.7	ND	ND	0.02	0.01	ND	0.03	61.2

^a^L_170_, L_180_, L_190_, L_200_, and L_210_ represent the lignin fractions obtained from the hydrothermal pretreated *Eucalyptus* at 170, 180, 190, 200, and 210 °C for 0.5 h followed by alkaline pretreatment with 2% NaOH aqueous solution at 80 °C for 2 h, respectively. L_0_ was fractionated from the untreated *Eucalyptus* directly under the same alkaline pretreatment condition.

^b^Based on the initial weight of the raw materials (%, w/w).

^c^Abbreviations: Ara, arabinose; Gal, galactose; Glc, glucose; Xyl, xylose; Man, mannose.

^d^Based on the dry mass of lignin (%, w/w).

^e^Yield of the hydrothermal pretreatment residue. The HTP residue yield was defined as g of hydrothermal pretreatment residue per 100 g of raw material on a dry weight basis.

^f^ND, not detectable.

To verify the purity of the lignin fractions, the associated carbohydrates of the lignin samples were analyzed by HPAEC. Clearly, all the lignin fractions contained rather low amounts of associated carbohydrates (0.03–0.32%), which was possibly due to the cleavage of chemical bonds between lignin and carbohydrates (LCC bonds) during the hydrothermal and alkaline pretreatments. In detail, xylose was the predominant sugar in lignin fractions, implying that the associated carbohydrates were mainly originated from xylans. An increase in pretreatment temperature from 170 to 210 °C led to a reduction of the associated carbohydrate contents from 0.32% to 0.03%, which was probably due to the cleavage of more LCC bonds under the harsh conditions. Moreover, the slight change of the glucose content indicated that cellulose was stable during the pretreatment. These results inferred that the integrated pretreatment process based on hydrothermal and alkaline pretreatments is an effective pretreatment technology to cleave the chemical bonds between lignin and carbohydrates, and thus can reduce the lignin content in biomass and increase the exposure of cellulose to enzymes, especially at 200 °C.

### Molecular weight analysis

Table 2 shows the weight-average (*M*_*w*_) and number-average (*M*_*n*_) molecular weights, as well as the polydispersity indices (*M*_*w*_/*M*_*n*_) of the lignin fractions. The obtained data can only be considered as relative molecular weights due to the calibration with polystyrene standards. As can be seen, the *M*_*w*_ and *M*_*n*_ of L_0_ were 3640 and 2760 g/mol, and its polydispersity index was 1.32. Since the cleavage of *β*-*O*-4′ linkages is one of the most important depolymerization reactions in lignin during the hydrothermal pretreatment, the molecular weights of the lignin fractions were expected to decline after hydrothermal pretreatment. However, when the temperature elevated from 170 to 200 °C, the *M*_*w*_ and *M*_*n*_ of the lignin fractions were increased from 4140 to 4610 and 3150 to 3640 g/mol, respectively. This could be explained by the fact that the cleavage of *β*-*O*-4′ linkages in lignin during hydrothermal pretreatment was accompanied with repolymerization reactions, resulting in an increase of molecular weights of the lignin fractions. The repolymerization reaction was due to acid-catalyzed condensation between the aromatic C6 or C5 and a carbonium ion, normally located at C_α_ of the side chain^23^. In contrast, the *M*_*w*_ and *M*_*n*_ of L_210_ were decreased to 3630 and 2780 g/mol as the temperature increased to 210 °C. This might be due to the fact that depolymerization reactions of lignin were dominant as compared with those of the repolymerization under the harsh condition. In addition, the six lignin fractions gave a relatively analogous polydispersity index, ranging from 1.27 to 1.31. It is well known that the lignin fraction with polydispersity index below three is indicative of molecularly uniform polymer and have potential applications in chemical industry^24^.

**Table 2:** Weight-average (*Μ*_*w*_) and number-average (*Μ*_*n*_) molecular weights and polydispersity indices (*Μ*_*w*_/*Μ*_*n*_) of the lignin fractions obtained from *Eucalyptus*.

Lignin fraction^a^	*Μ*_*w*_ (g/mol)	*Μ*_*n*_ (g/mol)	*Μ*_*w*_/*Μ*_*n*_
L_0_	3640	2760	1.32
L_170_	4140	3150	1.31
L_180_	4150	3170	1.31
L_190_	4240	3270	1.30
L_200_	4610	3640	1.27
L_210_	3630	2780	1.31

^a^Corresponding to the lignin fractions in Table 1.

### 2D-HSQC NMR spectral analysis

**Figure 1:**
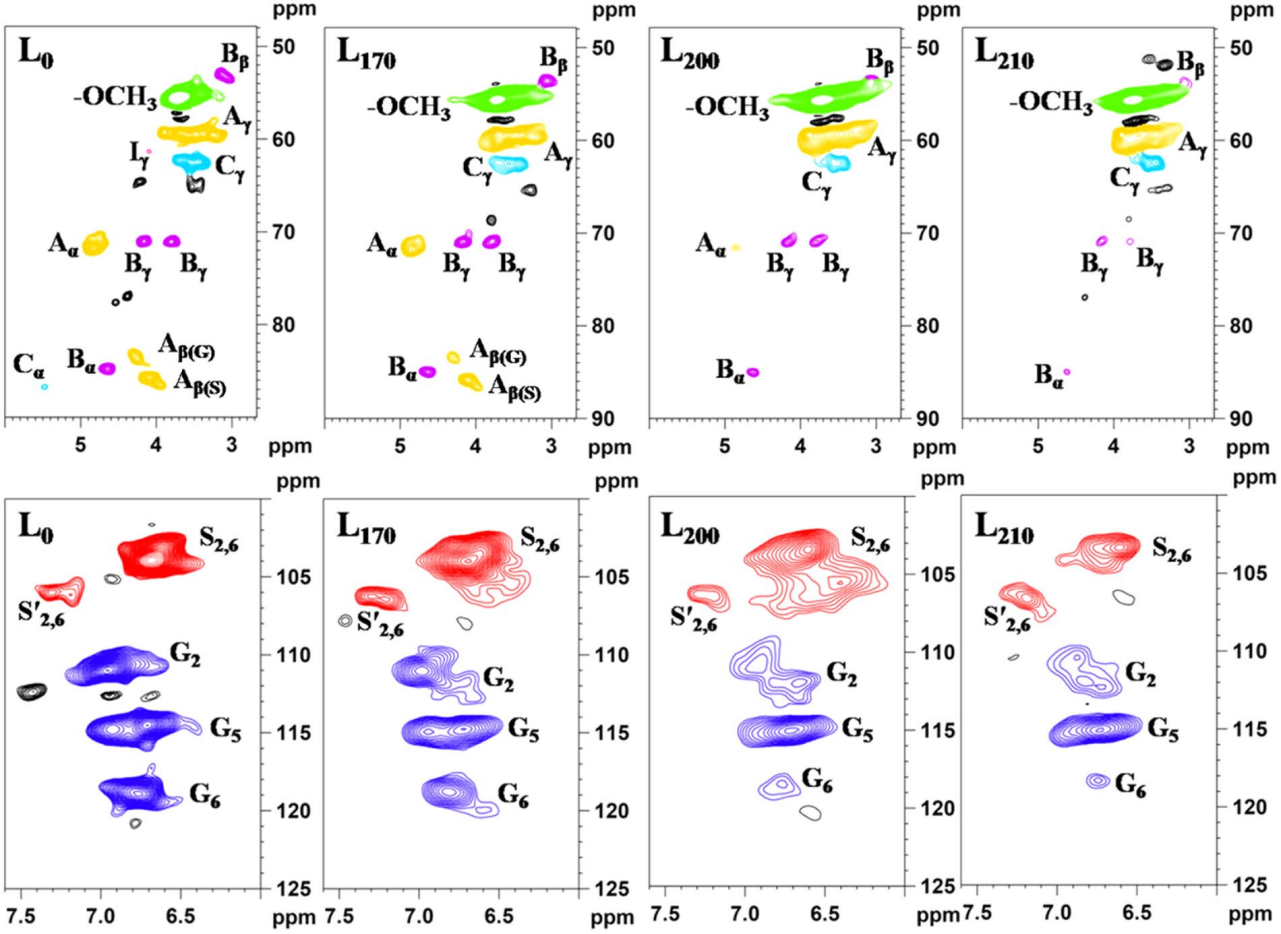
2D-HSQC spectra of the lignin fractions (L_0_, L_170_, L_200_, and L_210_) extracted from *Eucalyptus*.

2D-HSQC NMR spectroscopy is a powerful tool to provide better qualitative and semi-quantitative analysis as compared to the traditional chemistry methods. In the present study, the representative lignin fractions L_0_, L_170_, L_200_, and L_210_ were comparatively investigated by 2D-HSQC NMR technique. The HSQC NMR spectra of lignin can be divided into three regions: the aliphatic region, the side-chain region, and the aromatic region. The aliphatic region will not be discussed due to the lack of significant structural information of lignin in this region. The side-chain and aromatic regions of these lignin fractions are shown in Fig. 1, and the main substructures are represented in Fig. S1. Lignin contours in the 2D-HSQC spectra were annotated with peak assignments (Table S1) based on the previous literatures^25, 26^.

In the side-chain region of the spectra, the cross-signals of various interunit linkages and substructures of the lignin fractions were identified, such as *β*-aryl ethers (*β*-*O*-4′, A), resinols (*β*-*β*′, B), phenylcoumarans (*β*-5′, C), and non-acylated *p*-hydroxycinnamyl alcohol end groups (I). As can be seen, the cross-signals of methoxyl groups (−OCH_3_, *δ*_C_/*δ*_H_ 55.6/3.73) and *β*-*O*-4′ aryl ether linkages are the predominant substructures. Specifically, the C_*α*_–H_*α*_ correlations (A_*α*_) in *β*-*O*-4′ substructures were located at *δ*_C_/*δ*_H_ 71.8/4.83, while the C_*γ*_–H_*γ*_ correlations (A_*γ*_) were observed at *δ*_C_/*δ*_H_ 59.4/3.67. The C_*β*_–H_*β*_ correlations at *δ*_C_/*δ*_H_ 83.4/4.27 and 85.7/4.09 were linked to G (A_*β*(G)_) and S (A_*β*(S)_) units in *β*-*O*-4′ substructures, respectively. The presence of resinol (*β*-*β*′, B) substructures was confirmed by C–H correlations for *α*-, *β*- and *γ*-C positions centered at *δ*_C_/*δ*_H_ 84.8/4.64, 53.5/3.12, and 71.0/4.16 and 3.80, respectively. The lignin phenylcoumarans (*β*-5′, C) substructures were also detected in a relatively low level. The signals of their C_*α*_–H_*α*_ correlations were observed at *δ*_C_/*δ*_H_ 86.8/5.47, and the C_*γ*_–H_*γ*_ correlations were overlapped with other signals at *δ*_C_/*δ*_H_ 62.4/3.43. In addition, a minor amount of C_*γ*_–H_*γ*_ correlations in *p*-hydroxycinnamyl alcohol end groups (I) was discovered at *δ*_C_/*δ*_H_ 61.3/4.09.

The cross-signals of the lignin fractions for correlations in syringyl (S/S′) and guaiacyl (G) units were distinctly differentiated in the aromatic region. However, *p*-hydroxyphenyl (H) lignin units were not observed in any of the spectra. Obviously, the normal S-type lignin units showed a prominent signal for the C_2,6_–H_2,6_ correlation at *δ*_C_/*δ*_H_ 104.0/6.68, and the signals corresponding to C_2,6_–H_2,6_ correlations in C_*α*_-oxidized S units (*δ*_C_/*δ*_H_ 106.0/7.31) were also presented in the HSQC spectra. Moreover, the G units exhibited various correlations for C_2_–H_2_ (*δ*_C_/*δ*_H_ 111.0/6.96), C_5_–H_5_ (*δ*_C_/*δ*_H_ 114.5/6.71), and C_6_–H_6_ (*δ*_C_/*δ*_H_ 118.9/6.77), respectively. The double C_5_–H_5_ signals revealed some heterogeneity in the G units especially affecting the C_5_–H_5_ correlation, probably due to the different substituents at C_4_ (e.g., phenolic or etherified in different substructures). It should be noted that besides the normal signals for aromatic rings, some condensed S and G units appeared in the HSQC spectra. The C–H correlations from condensed lignin structures are the 2- and 6-positions of guaiacyl units linked at the 5-position to other lignin side chains^27^. However, the detailed structures of condensed lignin formed in S units remained unknown. The condensed position in S units is probably located at C_*α*_ of the side chain, which can react with another lignin unit and generate a new stable carbon–carbon linkage between two lignin units^23^.

Semi-quantification of the lignin fractions by 2D-HSQC NMR can provide precise structural evolution during the pretreatment. The relative abundances of interunit linkages and S/G molar ratios existing in L_0_, L_170_, L_200_, and L_210_ were investigated semi-quantitatively according to a previous method^28, 29^, and the values are listed in Table 3. As expected, the *β*-*O*-4′ linkages appeared to be the most abundant linkages in the lignin, followed by the resinols (*β*-*β*′) and lower amounts of the phenylcoumarans (*β*-5′). The content of *β*-*O*-4′ in L_0_ was 59.6/100Ar, while it decreased to 40.5/100Ar in L_170_. When the temperature exceeded 200 °C, the signal of *β*-*O*-4′ linkages was almost undetectable, indicating that the cleavage of *β*-*O*-4′ linkages was the predominant reaction during the hydrothermal pretreatment. Additionally, the content of *β*-*β*′ linkages was reduced as the temperature increased and the *β*-5′ linkages were cleaved completely when the temperature reached 210 °C. During the pretreatment, the depolymerization reactions of lignin were accompanied with repolymerization reactions as described in the molecular weight section. However, the contents of *β*-*O*-4′, *β*-*β*′, and *β*-5′ linkages were decreased with the increase of temperature. This may be due to the formation of more carbon–carbon linkages during the repolymerization reactions, which were hard to detect by NMR. Besides *β*-*O*-4′ aryl ether and carbon–carbon linkages, changes of the S/G ratio were also prominent during the hydrothermal pretreatment. The S/G ratio of L_170_ was 3.15, which was higher than that of L_0_. The increased S/G ratio might be as a consequence of the prior degradation of low molecular weight of non-condensed G-type lignin at the lower temperature^30, 31^. However, the S/G ratio gradually declined as the temperature increased to 210 °C, implying that the S-type lignin was more easily removed under the harsh HTP conditions. This is probably because the *β*-*O*-4′ linkages of S-type lignin were more reactive than those presented in G-type lignin under the higher temperature, making it comparatively easier to remove the S-type lignin^32^.

**Table 3:** Semi-quantification of the lignin fractions L_0_, L_170_, L_200_, and L_210_ obtained from *Eucalyptus* by semi-quantitative 2D-HSQC NMR.

	*β*-*O*-4′^a^	*β*-*β*′^a^	*β*-5′^a^	S/G^b^
L_0_	59.6	13.6	1.7	1.14
L_170_	40.5	12.6	1.7	3.15
L_200_	2.2	4.7	1.8	2.10
L_210_	Tr^c^	2.3	ND^d^	1.12

^a^Results expressed per 100 Ar based on semi-quantitative 2D-HSQC spectra. The C_*α*_–H_*α*_ correlations at *δ*_C_/*δ*_H_ 71.8/4.83 (A_*α*_), *δ*_C_/*δ*_H_ 84.8/4.64 (B_*α*_), and *δ*_C_/*δ*_H_ 86.8/5.47 (C_*α*_) were used for the integration to calculate the percentages of *β*-*O*-4′, *β*-*β*′, and *β*-5′ linkages, respectively.

^b^S/G ratio obtained by the equation: S/G ratio = 0.5*I* (S_2,6_)/*I* (G_2_).

^c^Tr, trace.

^d^ND, not detectable.

### ^31^P NMR spectral analysis

To further investigate the structure changes of lignin during the hydrothermal and alkaline pretreatments, quantitative ^31^P NMR technique was applied to detect the types and amount of hydroxyl groups in the lignin fractions. Table 4 exhibits the quantitative data on the distribution of the diverse hydroxyl groups as determined by the ^31^P NMR spectra (Fig. S2). The ^31^P NMR analysis confirmed that the original *Eucalyptus* lignin belongs to the syringyl–guaiacyl type, as evidenced by the presence of S-type and G-type phenolic OH in the ^31^P NMR spectra of all lignin fractions. It was observed that the content of aliphatic OH of the lignin fractions gradually reduced from 4.83 to 1.02 mmol/g as the pretreatment temperature increased. This may be partly attributed to the loss of the *γ*-methylol groups as formaldehyde and hydroxyl groups on C_α_ to form ketone structure^33^. In addition, the oxidization reactions of aliphatic OH might occur during the pretreatment, as supported by the slight increment of the carboxylic OH content (Table 4). The increased contents of phenolic OH from 0.94 (L_0_) to 2.87 (L_200_) mmol/g were resulted from the cleavage of *β*-*O*-4′ linkages, which can be confirmed by the lower content of *β*-*O*-4′ linkages calculated from 2D-HSQC spectra. However, the total phenolic OH decreased when the temperature further increased to 210 °C, which might be due to the condensation reactions occurred at an elevated temperature during the hydrothermal pretreatment. In addition, for L_0_, the content of S-type OH (0.29 mmol/g) was less than that of the G-type OH (0.65 mmol/g), which was possible that most of the S-type lignin units were involved in the formation of *β*-*O*-4′ linkages and only a small amount of free S-type OH could be detected by the ^31^P NMR spectra. Moreover, it was also observed that the increment of the S-type OH was more than the G-type OH as the temperature elevated to 200 °C, which further indicated that the *β*-*O*-4′ linkages were mainly composed of S-type lignin units.

**Table 4:** Hydroxyl concentrations (mmol/g) of the lignin fractions L_0_, L_170_, L_200_, and L_210_ obtained from *Eucalyptus* as determined by ^31^P NMR.

	Aliphatic OH	Syringyl OH	Guaiacyl OH	Carboxylic group	Total phenolic OH
L_0_	4.83	0.29	0.65	0.11	0.94
L_170_	2.78	0.87	0.74	0.31	1.61
L_200_	1.15	1.67	1.20	0.32	2.87
L_210_	1.02	0.93	0.96	0.53	1.89

### Confocal raman microscopy analysis

CRM can be used to provide label-free and nondestructive visualization of the histochemical changes in *Eucalyptus* at a subcellular level. The combination of chemical sensitivity with high spatial resolution made it possible to compare the signal intensity and distribution of lignin, and lignin could be imaged by integrating characteristic Raman bands^17, 34^. Figure S3 shows the bright field images of the samples tested. The band at 1598 cm^−1^ attributed to aromatic ring stretching is a general Raman signal of lignin. Another predominant band of lignin was detected at 1654 cm^−1^, which was due to the coniferaldehyde and coniferyl alcohol units^35^. There are two ways to achieve the Raman images of lignin distribution: using only the band at 1598 cm^−1^ and using both the bands at 1598 and 1654 cm^−1^. The Raman images obtained by them are almost identical, except that the second one that generates a better signal-to-noise ratio^36^. Therefore, the images of lignin distribution were acquired by integrating the combined band region of 1547–1707 cm^−1^.

**Figure 2:**
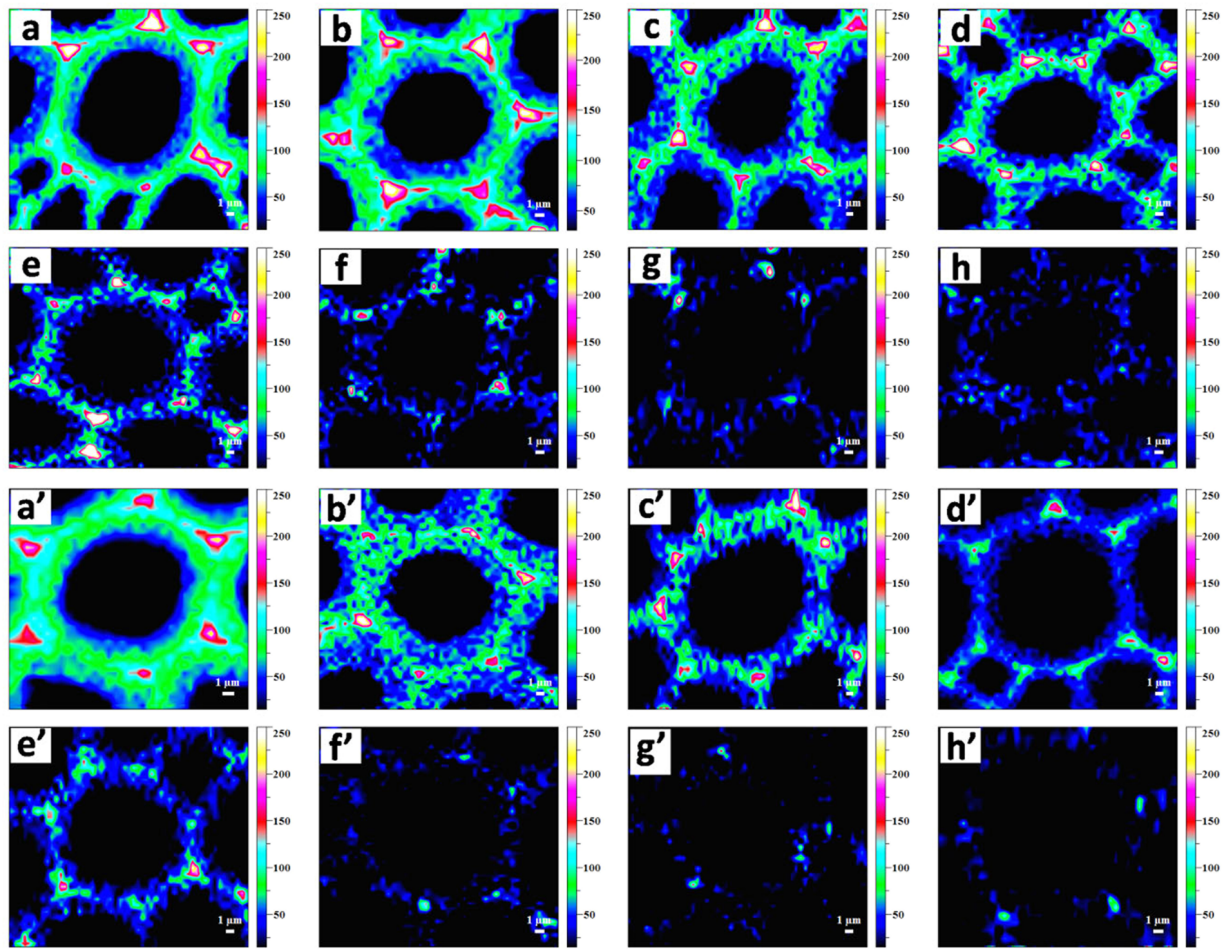
(**a**–**h**) Raman images of the lignin distribution (1547–1707 cm^−1^) in the *Eucalyptus* cell wall before and after hydrothermal pretreatment at 150, 160, 170, 180, 190, 200, and 210 °C for 0.5 h, respectively; (**a**′–**h**′) Raman images of the lignin distribution (1547–1707 cm^−1^) in the untreated and hydrothermally pretreated *Eucalyptus* cell wall further extracted with 2% NaOH at 80 °C for 2 h.

The lignin distributions in the *Eucalyptus* cell walls before and after hydrothermal pretreatment at 150, 160, 170, 180, 190, 200, and 210 °C for 0.5 h were investigated (Fig. 2(a–h)). For the untreated sample (Fig. 2(a)), the highest lignin concentration was observed in the cell corner middle lamella (CCML) regions (from black to white: intensity from low to high), followed by compound middle lamella (CML), and the lowest level of the intensity was in the secondary wall regions. After hydrothermal pretreatment, the pattern of lignin distribution displayed a similar trend with a reduction of the lignin signal intensity in different degrees. The strong contrast of intensity among morphologically distinct regions in the pretreated samples was primarily due to the different rates of delignification within various cell wall layers. As compared with the untreated sample, the hydrothermal pretreatment at 150, 160, and 170 °C resulted in a gradual reduction of lignin in the whole regions. As the temperature increased from 180 to 200 °C, the concentration of lignin was mainly reduced in the secondary wall regions; however, the samples did not show a substantial decrease of lignin in the CCML regions. This suggested that the dissolution of lignin was prominent in the S layers than that in the CCML regions. One possible explanation for this phenomenon is the penetration mechanism that the solvent allowed penetration into the cell walls from the lumen to the CCML and thus a preferential delignification occurred in the secondary wall regions^37^. Another reason could be that the reactivity of the varied lignin units in different morphological regions was discrepant. Previous studies demonstrated that the syringyl units with less branching were prominent in the secondary wall regions, which may result in a looser structure with fewer cross-links between the two units, and thus the secondary wall regions were easier to digest during the pretreatment^38, 39^. When the temperature further increased to 210 °C, the signal of the lignin almost disappeared, indicating a substantial removal of lignin.

The Raman images of the distributional changes of lignin in the untreated and hydrothermally pretreated cell walls extracted with 2% NaOH at 80 °C for 2 h were also shown in Fig. 2(a′–h′). The signal intensity of lignin after hydrothermal and alkaline pretreatments was lower than that of the only hydrothermally pretreated samples, indicating that alkaline pretreatment further led to the dissolution of lignin from cell walls. As for the sample only treated with NaOH (Fig. 2(a′)), the distribution pattern of lignin was similar to that of the untreated sample (Fig. 2(a)), showing an unconspicuous reduction of lignin in the whole regions. After hydrothermal pretreatment at 150 and 160 °C combined with alkaline pretreatments, an obvious reduction of signal intensity of lignin was observed in the secondary wall regions, while lower degree of delignification was observed in the CCML regions. As the temperature further increased from 170 to 210 °C, the signal of lignin both in the secondary wall and CCML regions was significantly depleted to be almost undetectable. The increased rate of delignification in the CCML regions was presumably caused by the raised penetration of reagents into CCML under the harsh conditions. As seen in Fig. 2(h′), the weak signal of lignin was still detected in the cell walls when temperature raised to 210 °C, suggesting that a part of lignin matrix was still tightly linked together after hydrothermal and alkaline pretreatments, due to the limited penetration and dissolution of cell walls^40^.

**Figure 3:**
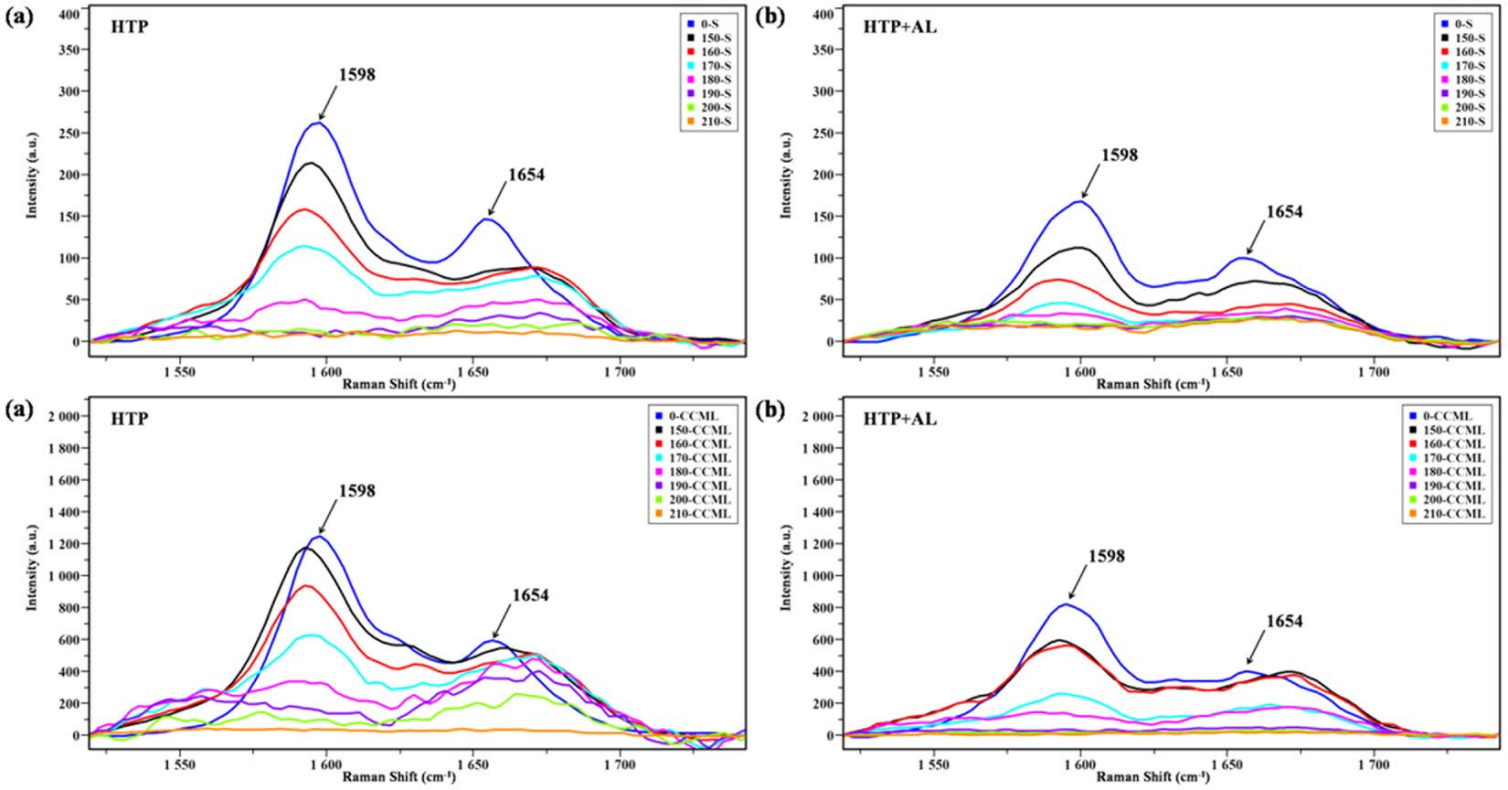
Zoom into average Raman spectra obtained from the secondary wall and CCML regions of *Eucalyptus* cell walls at different pretreatment conditions (**a**), hydrothermal pretreatment; (**b**), hydrothermal pretreatment followed by alkaline pretreatment, 1520–1742 cm^−1^. S, secondary cell wall; CCML, cell corner middle lamella.

To semi-quantitatively determine subtle dynamic changes in different morphological regions under various pretreatment conditions, a set of average Raman spectra obtained from the secondary wall and CCML regions are shown in Fig. 3. For both regions, the signal intensity of lignin displayed a reduction after the combined pretreatments, confirming the dissolution of lignin. As shown in Fig. 3(a), the dissolution of lignin in the secondary wall and CCML regions was unconspicuous as temperature increased from 150 to 170 °C. It should be noted that when temperature further increased to 210 °C, the band intensity was decreased significantly in the secondary wall regions and the lignin signal almost vanished at 210 °C. However, the decrease rate of lignin in the CCML regions was slower than that in the secondary wall regions. As illustrated in Fig. 3(b), the magnitude of reduction in the secondary wall regions was much higher than that in the CCML regions at the lower temperature from 150 to 180 °C. When the temperature further increased to 210 °C, the lignin concentration was decreased significantly both in the secondary wall and CCML regions. On the whole, the results indicated that the delignification rate was different within morphologically distinct regions and the preferential delignification occurred in the secondary wall regions.

### SEM analysis

**Figure 4:**
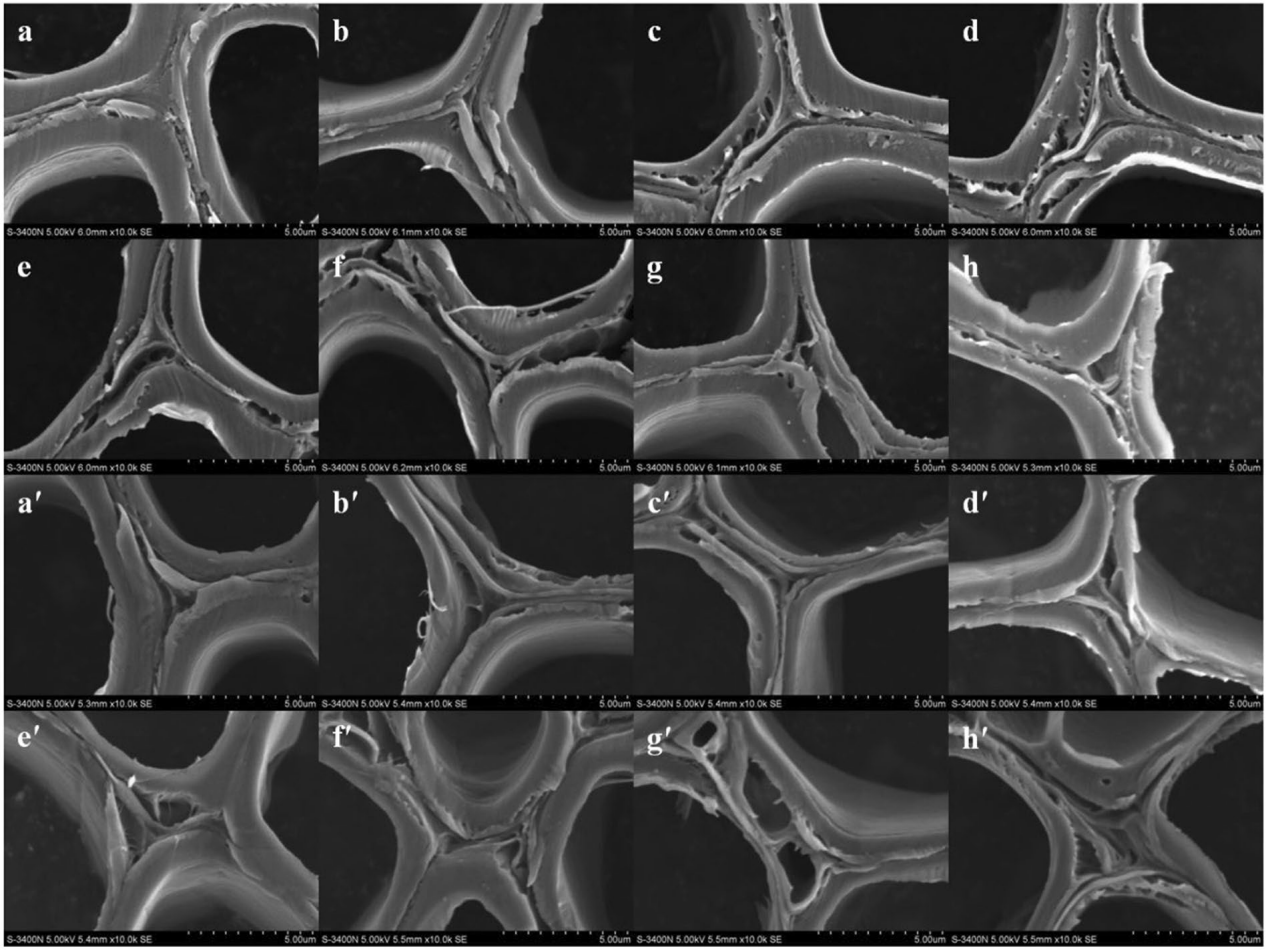
SEM images of the *Eucalyptus* cell walls at magnification × 10000: untreated (**a**), hydrothermally pretreated (at 150, 160, 170, 180, 190, 200, and 210 °C for 0.5 h, **b**–**h**), untreated and hydrothermally pretreated followed with 2% NaOH at 80 °C for 2 h (**a**′–**h**′).

The SEM images of the untreated and pretreated *Eucalyptus* sections obtained at magnification × 10000 are illustrated in Fig. 4. The results showed that the untreated sample (Fig. 4a) had a compact and relatively smooth cell wall surface structure, which could impede the accessibility of cellulase to cellulose. The cell wall was destroyed during the hydrothermal pretreatment with the formation of cracks on the biomass surface (Fig. 4b–h), especially at the boundary between CCML and secondary wall regions, which would benefit enzymatic digestibility of the pretreated materials. This may be due to the partial removal of hemicelluloses and lignin during the hydrothermal pretreatment. The cell walls of the section pretreated with hot water at 150 °C (Fig. 4b) were slightly damaged due to the limited impact of the pretreatment under the mild conditions. The disruption degree of cell walls was anabatic with the increased temperature (Fig. 4c–h). Under the most severe condition (Fig. 4h), the SEM image showed well-broken structure and an increased surface area as compared with the untreated sample. In addition, the SEM images revealed a range of spherical droplets that appeared on the cell walls at higher temperatures (Fig. 4g,h). These spherical droplets were thought to be mainly composed of lignin based on a previous report^21^. It seems clear that the hydrothermal pretreatment that exceeded the melting temperature of lignin allowed them to coalesce into larger molten bodies and redeposited as droplets on the surface of cell walls.

After hydrothermal pretreatment, the cell walls were further destroyed by the alkaline pretreatment, and the SEM images are shown in Fig. 4(a′–h′). The raw material merely treated with NaOH showed obvious cracks on the cell walls (Fig. 4a′), and the cracks were more evident as temperature increased (Fig. 4b′–h′). This was mainly explained by the removal of hemicelluloses and lignin during the alkaline pretreatment. When the temperature increased to 160 and 170 °C, the surface structure of the cell walls (Fig. 4c′,d′) tended to be smoother than those of the hydrothermally pretreated samples (Fig. 4c,d). After the pretreatment at 200 and 210 °C (Fig. 4g′,h′), the spherical droplets were disappeared from the cell wall, resulting in the exposure of more fiber bundles. Therefore, the integrated pretreatment process based on hydrothermal pretreatment followed by alkaline pretreatment is a promising technique to remove lignin from the cell wall and cellulose becomes more accessible and amenable to enzymatic hydrolysis.

## Conclusion

In summary, the combination of hydrothermal and alkaline pretreatments was used to treat *Eucalyptus*. It was found that the chemical bonds between lignin and hemicelluloses were cleaved to some extent, and thus can reduce the lignin content in biomass. The molecular weight analysis revealed that the cleavage of *β*-*O*-4′ linkages in lignin during hydrothermal pretreatment was accompanied with repolymerization reactions. Meanwhile, the NMR analysis suggested that an increasing temperature of the hydrothermal pretreatment resulted in the dropping content of *β*-*O*-4′, *β*-*β*′, and *β*-5′ linkages and aliphatic OH. The changes of lignin microdistribution were detected *in situ*, and the variations of lignin Raman intensity were more prominent in the secondary wall regions than that in the CCML regions. Overall, these results provided insights into the structure and spatial distribution changes of the lignin during the integrated pretreatment process, and thus enhance the understanding of the cell wall deconstruction during the pretreatment.

## Materials and Methods

### Materials

*Eucalyptus grandis* × *E*. *urophylla* wood, five years old, was collected from Guangxi province, China. It was dried, milled and sieved to select powders with sizes of 40–60 mesh. The powder was extracted with toluene–ethanol (2:1, v/v) in a Soxhlet apparatus for 6 h and then dried before use. The composition of the dewaxed material was 39.5% cellulose, 21.5% hemicelluloses, and 29.5% lignin (25.5% acid-insoluble lignin and 4.0% acid-soluble lignin). All chemicals were analytical grade and purchased from Sigma Chemical Company.

### Hydrothermal and alkaline pretreatments

The fractionation process for the lignin fractions from *Eucalyptus* is shown in Fig. S4. The *Eucalyptus* wood meal (3.0 g) was dispersed in 45 mL of distilled water in a reactor (Sen Long Instruments Company, Beijing, China). Then the reactor was heated to 150, 160, 170, 180, 190, 200, and 210 °C and kept for 0.5 h, respectively. Once the desired operation reached, the reactor was cooled down immediately and the mixtures were filtrated. The obtained solid residues were dried after thoroughly washed with distilled water and then extracted with 2% NaOH at 80 °C for 2 h with a solid to water ratio of 1:20 (g/mL). The filtrates were neutralized to pH 5.5–6.0 with HCl, and concentrated to about 30–50 mL. Subsequently, each concentrated solution was added into three volumes of 95% ethanol with constant stirring. The residual supernatants were poured into 5 volumes of distilled water after evaporation of ethanol, and the pH was adjusted to 1.5–2.0 with HCl to precipitate lignin fractions. The lignin obtained was freeze-dried and labeled as L_170_, L_180_, L_190_, L_200_, and L_210_. The control lignin fraction L_0_ was also obtained from the raw material under the same alkaline pretreatment condition without hydrothermal pretreatment.

For Raman spectroscopy, small *Eucalyptus* blocks of approximately 1 cm × 0.5 cm × 2 cm were cut from the stem manually. Cross sections of 10 μm thickness were prepared on a sliding microtome (Leica Microsystems, wetzlar, Germany). 10 *Eucalyptus* cross sections were placed in a reactor with 10 mL ultrapure water, and then heated to 150, 160, 170, 180, 190, 200, and 210 °C for 0.5 h, respectively. At the end of HTP, the reactor was cooled down immediately to room temperature. The untreated and hydrothermally pretreated sections were treated with 2% NaOH at 80 °C for 2 h. The reactor was cooled down immediately after the pretreatment and the sections were thoroughly washed with ultrapure water. Then the sections were stored in ultrapure water at 4 °C for further analysis.

### Analysis procedures

The content of the associated carbohydrates and the molecular weights of the lignin fractions were determined as described previously^41^. The 2D-HSQC NMR spectra and quantitative ^31^P NMR spectra of the lignin fractions were recorded on a Bruker AVΙΙΙ 400 MHz spectrometer at 25 °C. For 2D-HSQC spectroscopic experiments, the data were acquired in HSQC mode using 40 mg of lignin in 0.5 mL of dimethylsulfoxide-*d*_6_ (DMSO-*d*_6_). ^31^P NMR spectra were acquired after the reaction of lignin with 2-chloro-4,4,5,5-tetramethyl-1,3,2-dioxaphospholane (TMDP) according to a previous publication^41^.

The Raman spectra of the untreated and pretreated sections were detected on a LabRAM Xplora exquisite full-automatic confocal Raman microscope (Horiba Jobin–Yvon, Longjumeau, France) according to a previous literature^42^. LabSpec 5 software was used for image processing and spectral analysis. The spectra were baseline corrected with the Savitsky–Golay algorithm for spectroscopic analysis. The surface morphology of the untreated and pretreated *Eucalyptus* sections was detected using S-3400N (HITACHI, Japan) scanning electron microscope with a secondary electron detector. Prior to acquire images, all the samples were prepared for imaging by vacuum drying and then coated with a thin layer of gold in a sputter coater (E-1010, HITACHI, Japan). Imaging was performed with a 10 kV accelerating voltages at magnification × 10000.

## Electronic supplementary material

Supporting information
