# Supplementary material for: Revealing the structure and distribution changes of Eucalyptus lignin during the hydrothermal and alkaline pretreatments
Source: Sci Rep. 2017 Apr 4;7:593. doi: 10.1038/s41598-017-00711-w (PMC5429616; doi:10.1038/s41598-017-00711-w)
Supplement: Supplementary file 1 — Supporting information [file 41598_2017_711_MOESM1_ESM.pdf]

Supporting Information

Revealing the structure and distribution changes of *Eucalyptus* lignin during the hydrothermal and alkaline pretreatments

Chenzhou Wang[1], Hanyin Li[1], Mingfei Li[1], Jing Bian[1, *], Runcang Sun[1,2,*]

[1]Beijing Key Laboratory of Lignocellulosic Chemistry, Beijing Forestry University, Beijing 100083, China

[2]State Key Laboratory of Pulp and Paper Engineering, South China University of Technology, Guangzhou 510640, China

[*]Corresponding authors at: Beijing Key Laboratory of Lignocellulosic Chemistry, Beijing Forestry University, Beijing 100083, China. Tel.: +86 10 62336592; Fax: +86 10 62336903.

E-mail address: bianjing31@bjfu.edu.cn (J. Bian); rcsun3@bjfu.edu.cn (R.C. Sun).

Table S1

Chemical shifts and assignments of main ^{13}C–^{1}H cross-signals in the HSQC spectra of the lignin fractions extracted from *Eucalyptus*.

Lable	δ_C/δ_H (ppm)	Assignment
B_β	53.5/3.12	C_β–H_β in resinol substructures (B)
–OCH_3	55.6/3.73	C–H in methoxyls
A_γ	59.4/3.67	C_γ–H_γ in *β*-*O*-4′ substructures (A)
I_γ	61.3/4.09	C_γ–H_γ in *p*-hydroxycinnamyl alcohol end groups (I)
C_γ	62.4/3.43	C_γ–H_γ in phenylcoumaran substructures (C)
B_γ	71.0/3.80–4.16	C_γ–H_γ in resinol substructures (B)
A_α	71.8/4.83	C_α–H_α in *β*-*O*-4′ substructures (A)
$A_{\beta(G)}$	83.4/4.27	C_β–H_β in *β*-*O*-4′ substructures linked to G units (A)
B_α	84.8/4.64	C_α–H_α in resinol substructures (B)
$A_{\beta(S)}$	85.7/4.09	C_β–H_β in *β*-*O*-4′ substructures linked to S units (A)
C_α	86.8/5.47	C_α–H_α in phenylcoumaran substructures (C)
$S_{2,6}$	104.0/6.68	$C_{2,6}$–$H_{2,6}$ in syringyl units (S)
$S'_{2,6}$	106.0/7.31	$C_{2,6}$–$H_{2,6}$ in oxidized syringyl units (S′)
G_2	111.0/6.96	C_2–H_2 in guaiacyl units (G)
G_5	114.5/6.71	C_5–H_5 in guaiacyl units (G)
G_6	118.9/6.77	C_6–H_6 in guaiacyl units (G)

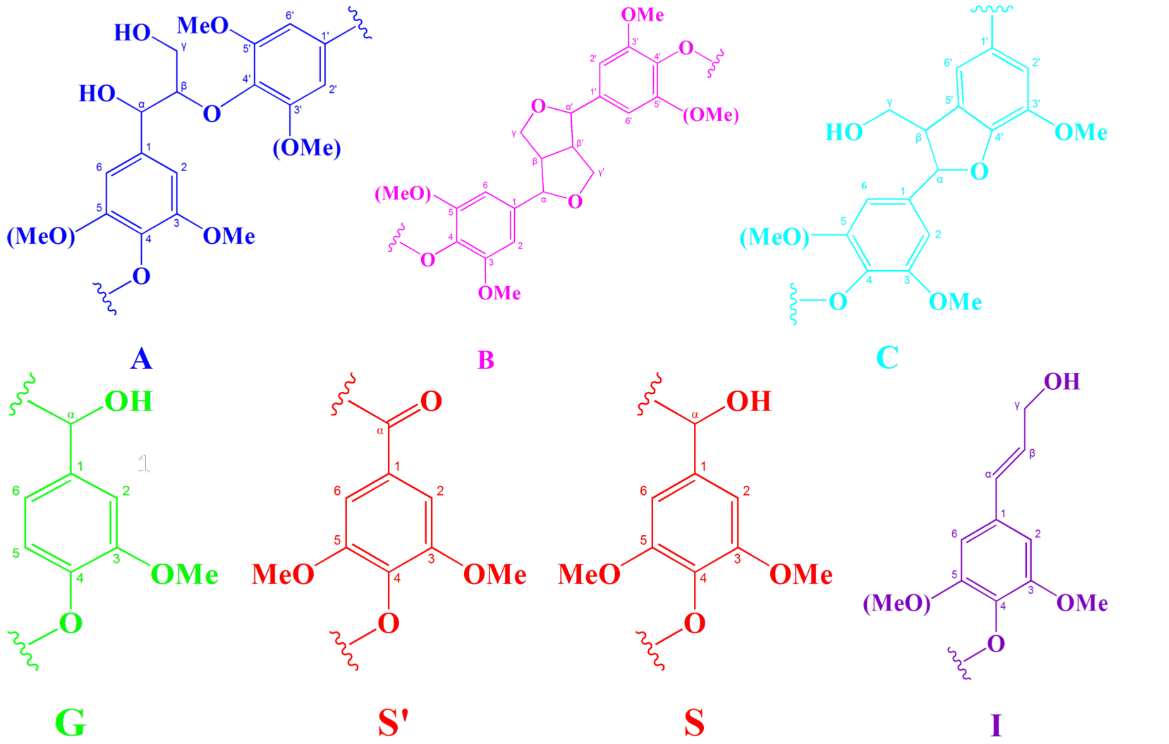

Fig. S1. The main substructures of the lignin fractions: (A) *β*-aryl-ether units (*β*-*O*-4′); (B) resinol substructures (*β*-*β*′); (C) phenylcoumaran substructures (*β*-5′); (G) guaiacyl units; (S′) oxidized syringyl units bearing a carbonyl at C_{α}; (S) syringyl units; (I) non-acylated *p*-hydroxycinnamyl alcohol end-groups.

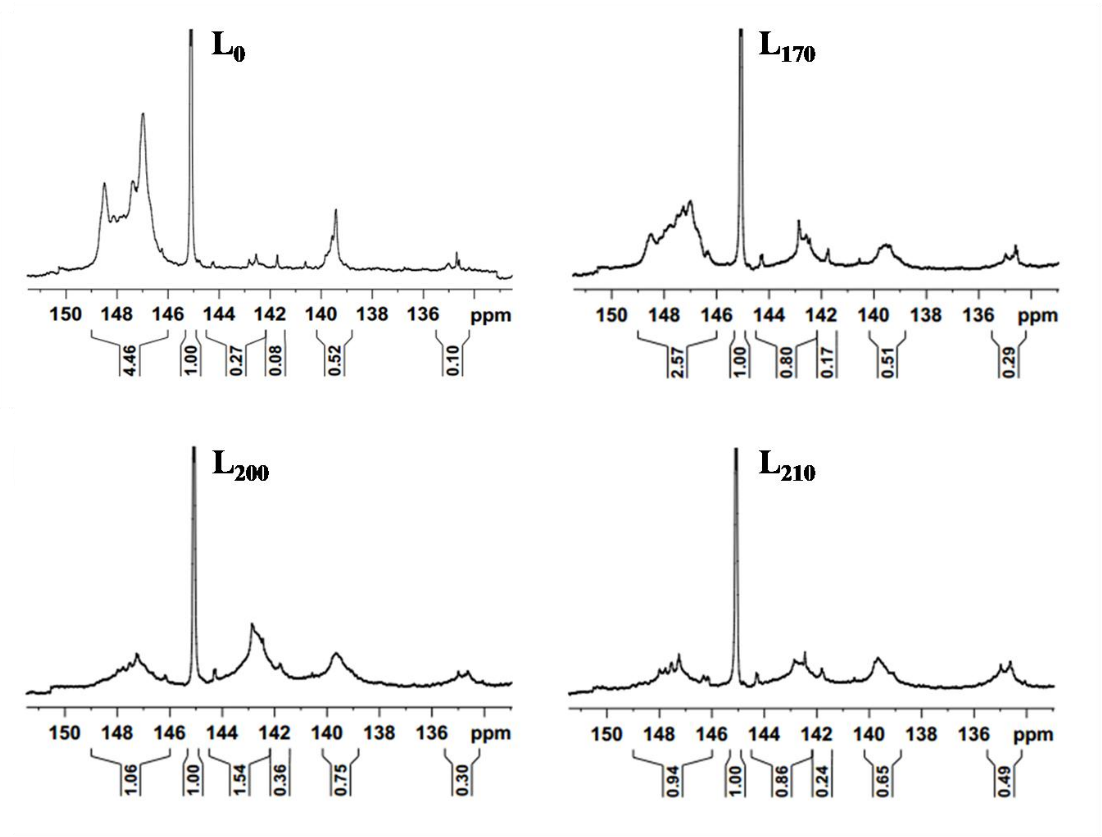

Fig. S2. ^{31}P-NMR spectra of the lignin fractions (L_0, L_{170}, L_{200}, and L_{210}) extracted from the untreated and hydrothermally pretreated *Eucalyptus* fibers.

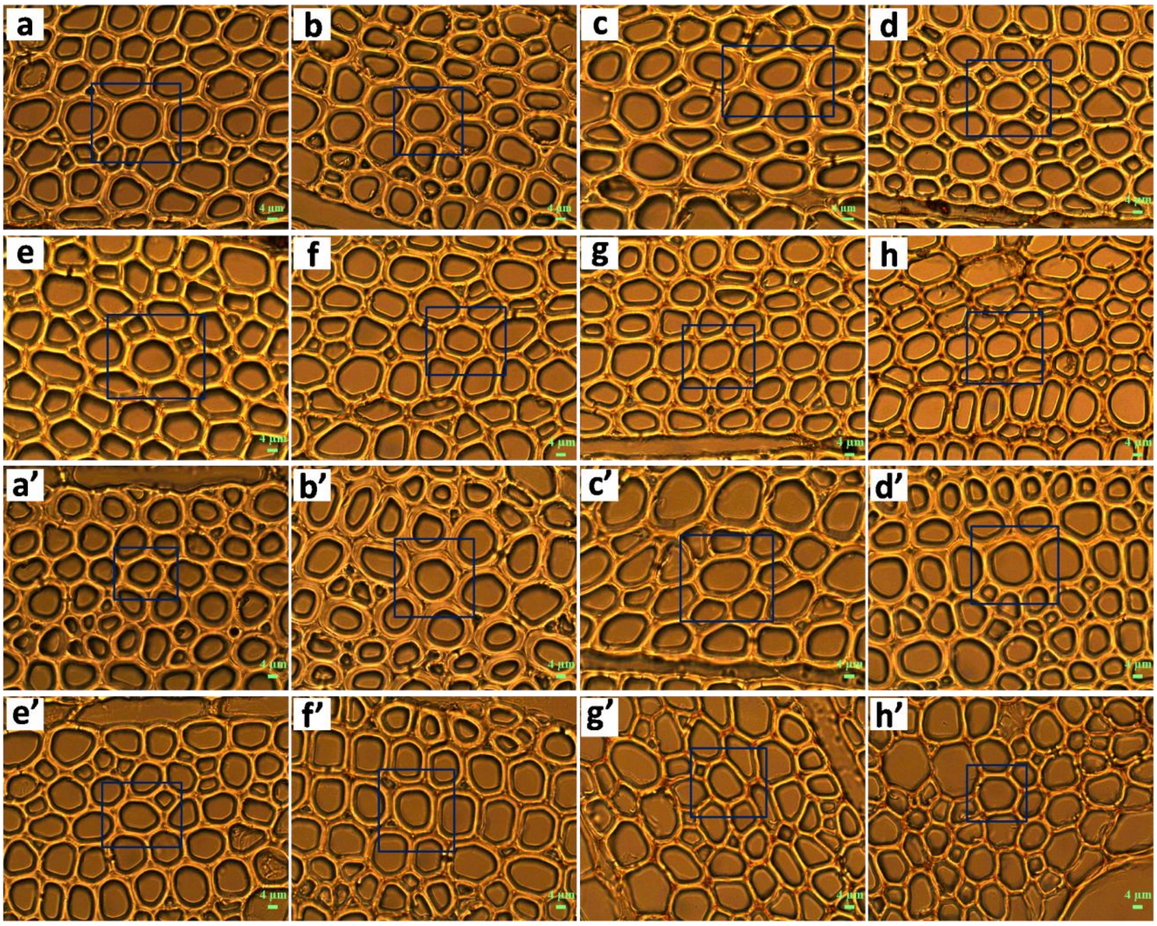

Fig. S3. a–h Bright field images of *Eucalyptus* cell wall before and after hydrothermal pretreatment at 150, 160, 170, 180, 190, 200, and 210 ℃ for 0.5 h, respectively; **a′–h′** bright field images of untreated and hydrothermally pretreated *Eucalyptus* cell wall further extracted with 2% NaOH at 80 ℃ for 2 h. Selected area (blue rectangle) shown in bright field images was used for Raman imaging.

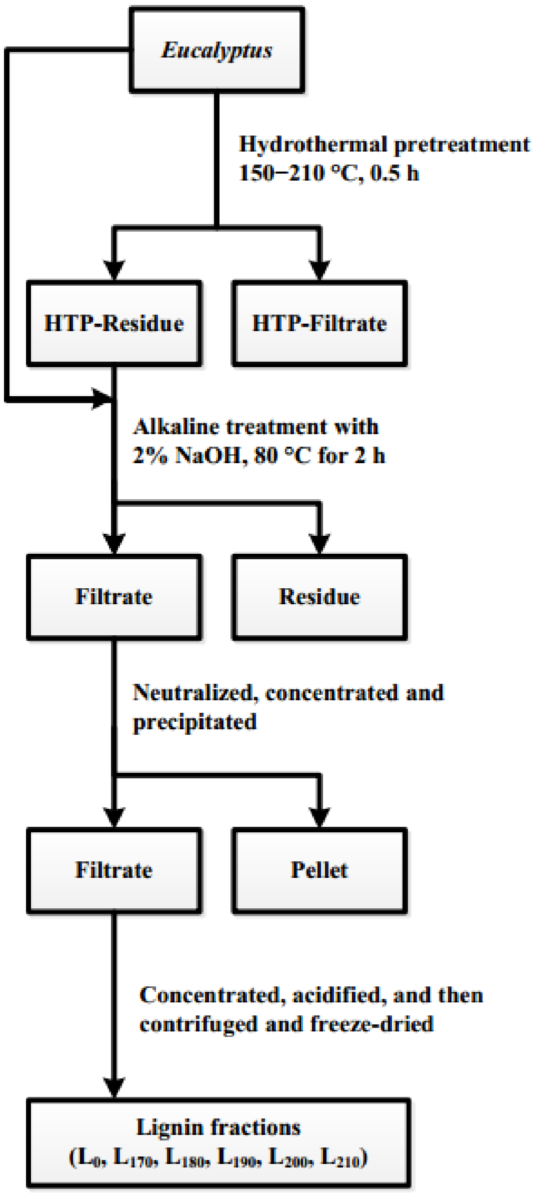

Fig. S4. Scheme for extraction of lignin fractions from *Eucalyptus*.
